# Lymphoproliferation and hyper-IgM as the first manifestation of activated phosphoinositide 3-kinase δ syndrome: A case report

**DOI:** 10.7705/biomedica.7436

**Published:** 2024-12-30

**Authors:** Mónica Fernandes-Pineda,, Andrés F. Zea-Vera

**Affiliations:** 1 Departamento de Medicina Interna, Universidad del Valle, Cali, Colombia Universidad del Valle Universidad del Valle Cali Colombia; 2 Departamento de Microbiología, Facultad de Salud, Universidad del Valle, Cali, Colombia Universidad del Valle Universidad del Valle Cali Colombia; 3 Genetic Immunotherapy Section, Laboratory of Clinical Immunology and Microbiology, National Institute of Allergy and Infectious Disorders, National Institutes of Health, Bethesda, MD, USA National Institutes of Health National Institutes of Health Bethesda USA

**Keywords:** Immune system diseases, phosphatidylinositol 3-kinase, hyper-IgM immunodeficiency syndrome, autoimmunity, human genetics, genetic testing, enfermedades del sistema immunológico, fosfatidilinositol 3-cinasa, síndrome de inmunodeficiencia con hiper-IgM, autoinmunidad, genética humana, pruebas genéticas

## Abstract

Activated phosphoinositide 3-kinase δ syndrome is an inborn error of immunity due to mutations within the genes responsible for encoding PI3Kδ subunits. This syndrome results in an excessive activation of the phosphoinositide 3-kinase signaling pathway. Gain- of-function mutations in the gene *PIK3R1* (encoding p85α, p55α, and p50α) lead to the development of the activated PI3K δ syndrome. Notably, the clinical presentations of this syndrome often closely resemble those of other primary immunodeficiencies.

We present a case involving a 15-year-old male who displayed an immunological phenotype that bore a striking resemblance to hyper-IgM syndrome. Whole exome sequencing was undertaken to pinpoint the underlying genetic mutation.

Our investigation successfully identified a heterozygous splice site mutation previously reported within the well-established hotspot of the *PIK3R1* gene (GRCh37, c.1425+1 G>T). The diverse spectrum of inborn errors of immunity underscores the pivotal role of identifying gene mutations, particularly in patients presenting clinical manifestations spanning autoimmune disorders, lymphoproliferative conditions, and antibody deficiencies. Such precise genetic diagnoses hold significant potential for improving patient care and management.

## Resumen

El síndrome de la fosfoinosítido 3-cinasa delta activada es un error innato de la inmunidad debido a mutaciones en los genes responsables de codificar las subunidades de la enzima PI3Kδ, lo que resulta en una activación excesiva de la vía de señalización de la fosfoinosítido 3-cinasa. Las mutaciones de aumento de función del gen *PIK3R1* -que codifica para ρ85α, p55a y p50a- conducen al desarrollo del síndrome de activación de PI3K delta 2. A menudo, las presentaciones clínicas de este síndrome se asemejan a las de otras inmunodeficiencias primarias.

Se presenta el caso de un paciente de sexo masculino de 15 años, que mostró un fenotipo inmunológico semejante al del síndrome de hiper-IgM. Para determinar la mutación genética subyacente, se llevó a cabo un análisis de secuenciación de exoma completo. En este estudio se identificó con éxito una mutación heterocigota in situ, reportada previamente dentro del hotspot del gen *PIK3R1* (GRCh37, c.1425+1 G>T). El diverso espectro de errores innatos de la inmunidad resalta la importancia de identificar mutaciones génicas, particularmente en pacientes que presentan manifestaciones clínicas, como trastornos autoinmunitarios, condiciones linfoproliferativas y deficiencias de anticuerpos. Los diagnósticos genéticos precisos tienen un potencial significativo para mejorar la atención y el manejo del paciente.

Class IA phosphatidylinositol 3-kinases (PI3K) constitute a vital family of heterodimeric enzymes consisting of a 110-kDa catalytic subunit (ρ110α, β, or δ) paired with a regulatory subunit (ρ85α, ρ85β, ρ55α, ρ50α, or ρ55γ). These PI3K enzymes play a pivotal role in immune cell activation, orchestrating essential functions such as cell growth, proliferation, survival, migration, and differentiation ^1^.

Genetic mutations in genes encoding PI3K subunits can lead to various immune-related disorders. Specifically, mutations in the PIK3R1 gene, responsible for encoding phosphatidylinositol 3-kinase regulatory subunits, underlie the condition known as activated phosphoinositide 3-kinase δ syndrome 2 (APDS2), while mutations in PIK3CD are associated with APDS1 ^2^.

The clinical manifestations of these syndromes encompass symptoms akin to those of other inborn errors of immunity, including recurrent bacterial respiratory infections, heightened susceptibility to herpes virus infections, lymphoproliferation, autoimmunity, enteropathy, and lymphoma ^3^.

In this case report, we present the clinical profile of a male patient harboring a PIK3R1 mutation, which has led to the manifestation of APDS2, characterized by hyper-IgM and lymphoproliferation, evident since childhood.

## Case report

A 15-year-old Colombian boy, born to non-consanguineous healthy parents, was the first child in the family with one unaffected sibling. His medical history revealed an uneventful vaccination with the Calmette- Guérin bacillus (BCG) at birth. At five months of age, he began experiencing recurrent upper respiratory tract infections. A subsequent episode of gastroenteritis at six months necessitated oral antibiotic treatment. At two years old, the patient was admitted to the emergency room due to the bronchoaspiration of a peanut, requiring bronchoscopy for removal.

During a routine physical examination, visible tonsils and bilateral submandibular and cervical lymphadenopathies (greater than 2 cm in diameter) were noted. However, other physical findings were unremarkable, with no abnormalities detected in the lungs, heart, abdomen, or nervous system. Upon reassessment, the patient’s mother reported a persistent high fever (> 38.5 °C), night sweats, and a chronic cough over the previous six months. Subsequently, the patient was admitted to the pediatric infectious disease service, where tuberculosis was ruled out, and additional investigations were conducted.

Serological testing revealed positive IgM and negative IgG for cytomegalovirus (CMV), while tests for Epstein-Barr (EBV) mononucleosis were negative. Viral load assessments for EBV or CMV were unavailable (table 1). A lymph node biopsy demonstrated lymphoid hyperplasia without evidence of malignancy. Infectious complications were considered, and due to suspected acute CMV infection and potential exposure to pets, cat scratch disease was suspected, leading to the prescription of oral antibiotics (azithromycin).

**Table 1:** Patient’s results of the laboratory tests

Laboratory parameter	Date 2011 (3 yo)	Date 2012 (4 yo)	Date 2015 (7 yo)	Date 2019 (11 yo)	Date 2022 (14 yo)	Reference values
Leukocyte count (cel/pl)	7,030	15,700			7,310	6,000 - 17,500 (3 yo)
Neutrophil count (cel/pl)	1,360	6,140			2,920	1,500 - 8,500
Lymphocyte count (cel/pl)	4,290	6,760			3,390	3,000 - 9,500
Hematocrit (%)	27.7	30.7			32.2	31 - 43 (3 - 14 yo)
Platelet count (cel/pl)	692,000	440,000			435,000	150,000 - 450,000
IgG (mg/dl)		< 320	787	415.34	480.51	700 - 1,600
IgA (mg/dl)		< 5	< 33	< 40	<40	70 - 400
IgM (mg/dl)		1,290	879.1	468.5	651.32	40 - 230
IgE (UI/ml)		15.5	0	< 0.100	< 0.100	< 200
B lymphocytes (CD19+) cel/pl (%)					169 (3.3)	200 - 600 (8 - 24)
T lymphocytes (CD3+) cel/pl (%)					3,775.5 (80.5)	1,088.1 - 2,087.9 (53.3 - 75.3)
T lymphocytes (CD3+CD4+) cel/pl (%)					963.0 (20.5)	639.5 - 1,278.5 (30.7 - 46)
T lymphocytes (CD3+CD8+) cel/pl(%)					2,198 (46.6)	377.1 - 876.9 (20.3 - 36.1)
NK cells (CD3-CD56+CD16+) cel/pl (%)					690 (13.6)	70 - 1,200 (6 - 27)
PPD (mm)					0	> 10
Toxoplasma IgG (UI/ml)	0					< 15
Toxoplasma IgM (UI/ml)	0.288					< 0.4
Monotest	Negative					
Citomegalovirus IgG (UI/ml)	0	0				< 15
Citomegalovirus IgM (UI/ml)	1.307	1.976				< 0.399
VIH (index)	0.37					< 1
AgSHBV (index)	0.17	0.75				< 1.2

NK: natural killer; PPD: purified protein derivative test; HIV: human immunodeficiency virus; AgSHBV: hepatitis B surface antigen

At three years of age, the patient was readmitted with suspected lymphoproliferative disease attributed to the enlargement of cervical nodules. Physical examination revealed persistent submandibular and cervical lymphadenopathy but no signs of hepatosplenomegaly. Bone marrow aspirate analysis showed a cellular composition comprising 5% megakaryocytes, 61% myeloid lineage, 31% lymphocytes, and 3% eosinophils, with no evidence of tumorous growth.

Serological assessments indicated persistently negative EBV and CMV IgG, yet CMV IgM remained consistently positive, with escalating titers. Serum immunoglobulin levels revealed elevated IgM (1,290 mg/ dl) with undetectable IgG and IgA, leading to the diagnosis of hyper- IgM immunodeficiency in 2012, according to the European Society for

Immunodeficiencies (ESID) criteria. Consequently, monthly intravenous immunoglobulin treatment (400 - 600 mg/kg) was recommended, along with the prophylactic use of macrolides (azithromycin). Despite these measures, lymphadenopathies persisted.

**Figure 1:**
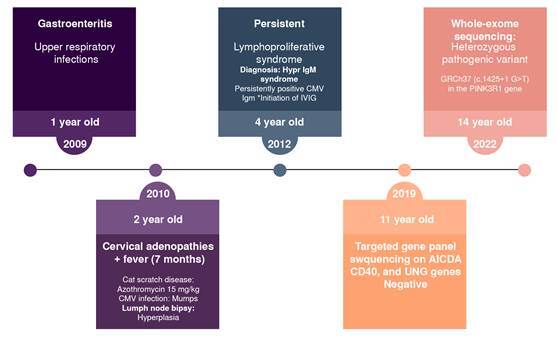
Clinical history timeline

In 2019, targeted gene panel sequencing for *AICDA, CD40, CD40L*, and *UNG* genes yielded negative results, failing to establish a molecular diagnosis during the clinical evaluation and follow-up. A renewed genetic assessment in 2022, when he was 14 years old, employing a comprehensive whole exome sequencing approach, revealed the heterozygous pathogenic variant c.1425+1G>T in the *PIK3R1* gene, confirming the diagnosis of APDS2 (figure 1).

CMV: cytomegalovirus; IV Ig: intravenous immunoglobulin

Currently, the individual receives monthly intravenous immunoglobulin substitution and is being closely observed for any changes in lung function. Recent scans showed no evidence of bronchiectasis or new masses. It is worth mentioning that the patient was last hospitalized at 11 years old due to pansinusitis. Since then, he has not had an additional decline in their health condition, although he still has persistent splenomegaly and adenomegaly.

### Ethical considerations

The authors obtained the written informed consent from the mother and the assent from the patient mentioned in the article. The corresponding author possesses this document.

## Discussion

We present the first published case of activated phosphoinositide 3-kinase δ syndrome 2 (APDS2) in Colombia, involving a young boy with a heterozygous splice site mutation in the PIK3R1 gene. The patient’s clinical presentations were chronic lymphadenopathy, recurrent viral and bacterial infections, and hepatosplenomegaly. This case underscores the diagnostic complexities often encountered in patients with APDS1/2, as the initial diagnosis of hyper-IgM syndrome gave way to a genetic diagnosis obtained through subsequent whole exome sequencing.

Phosphoinositide 3-kinases (PI3Ks) are integral enzymes within the PI3K-AKT-mTOR signaling pathway, playing a crucial role in the metabolism, differentiation, proliferation, growth, survival, and migration of immune cells ^4^. Mutations affecting the ΡΙ3Κδ regulatory subunit lead to the constant hyperactivation of the Akt-mTOR pathway in B and T lymphocytes. This hyperactivation -due to mutations in the *PIK3R1* gene coding for regulatory subunits- results in APDS2, marked by progressive lymphopenia and compromised differentiation and function of T and B cells ^5^.

APDS2 patients frequently suffer from multiple infectious complications, including recurrent upper and lower respiratory tract infections, such as sinusitis, otitis media, and pneumonia ^6^. These complications arise due to defects in antibody and cytokine production. Our patient experienced multiple hospitalizations for otitis media and sinusitis, which responded well to antibiotic therapy. Despite these recurrent respiratory infections, our patient did not exhibit bronchiectasis in the follow-up thorax computed tomography scan, sometimes developed by APDS2 patients after repeated respiratory infections ^7^.

Notably, our patient initially presented with CMV infections, ultimately leading to the diagnosis of the hyper-IgM phenotype. The literature has reported that APDS2 patients may experience recurrent infections caused by herpes family viruses, including EBV, CMV, herpes simplex virus, and varicella-zoster virus ^6^.

Beyond infectious complications, APDS2 patients can encounter non- infectious issues, including lymphadenopathy, hepatosplenomegaly, autoimmune and autoinflammatory disorders, malignancies, and growth problems. In our case, recurrent hospitalizations were attributed to cervical and submandibular lymphadenopathy, often linked to overactive proliferation in lymph node germinal centers, frequently triggered by EBV infections ^3^. Hepatosplenomegaly, observed in other APDS2 cases, was also evident in our patient ^8^. Moreover, the lymph nodes exhibited hyperplasia, and bone marrow biopsies ruled out hematological malignancies.

The common occurrence of the hyper-IgM phenotype in APDS2 patients may be a consequence of the dysregulation of PI3K signaling and classswitching recombination in developing B lymphocytes ^9^. Our patient’s immunoglobulin profile strongly suggested hyper-IgM syndrome despite negative results on molecular diagnostic tests for the *AICDA*, *CD40*, and *UNG* genes. Consequently, the clinical management of the patient was done according to the hyper-IgM syndrome diagnosis.

Notably, the variants localized at c.1425 of the *PIK3R1* gene are considered hotspot mutations and affect the donor splice site (c.1425+1G>A, C, T, c.1425+2T>G, A, c.1425+2,3delTG) and the acceptor splice site (c.1300-1G>C) of intron 10 ^4^. Consequently, exon 10 is skipped, leading to the ρ85α regulatory subunit truncation and the activation of PI3K signaling activity in APDS ^10^. Our patient harbored the previously reported heterozygous pathogenic variant c.1425+1G>T in the *PIK3R1* gene ^6^^,^^11^.

It is worth noting that APDS was first described in 2014, and since then between 47 and 100 new cases have been reported. Our patient exhibited symptoms in 2010, four years before the disease was described ^12^ (figure 1). The field of inborn errors of immunity is rapidly evolving, with new genes reported and new diseases defined annually ^13^. This field requires ongoing assessment of patients being studied for primary immunodeficiency. The importance of characterizing such orphan diseases has led to the development of broad population registries, like the European one, which includes 170 APDS patients and evaluates their heterogeneity ^14^.

In Latin America, increasing genetic diagnoses in patients with common variable immunodeficiency or hyper-IgM syndrome, whose etiology remains unknown, can potentially lead to therapeutic changes ^15^. Some inborn errors of immunity have specific treatments, such as leniolisib in APDS1 and APD2, in phase three clinical trials. Leniolisib, a ΡΙ3Κδ inhibitor, can mitigate long-term complications associated with lymphoproliferation and positively impact immune dysregulation in APDS patients ^16^. This treatment helps to decrease lymphadenopathy and splenomegaly, increase B lymphocyte counts, address cytopenias, and improve overall symptoms ^17^. This underscores the need for greater awareness and genetic testing in the Latin American context to improve patient care and management ^18^.

## Conclusion

Initially, the patient’s clinical immunoglobulin profile suggested hyper-IgM syndrome. However, inborn errors of immunity represent a dynamic field characterized by an ever-expanding array of newly described gene disorders. This evolving landscape underscores the importance of considering these novel gene disorders for patients without a molecular diagnosis.

Given the inherent heterogeneity within the spectrum of inborn errors of immunity, the identification of gene mutations becomes an invaluable tool in accurately diagnosing patients with clinical manifestations resembling autoimmune disorders, lymphoproliferative conditions, and antibody deficiencies. As our understanding of these conditions continues to evolve, early genetic diagnoses can significantly enhance patient care and management.
